# Self-Reported Oral Health, Oral Hygiene and Associated Factors in Lithuanian Adult Population, 1994–2014

**DOI:** 10.3390/ijerph17155331

**Published:** 2020-07-24

**Authors:** Asta Raskiliene, Vilma Kriaucioniene, Jolanta Siudikiene, Janina Petkeviciene

**Affiliations:** 1 Health Research Institute, Faculty of Public Health, Lithuanian University of Health Sciences, 47181 Kaunas, Lithuania; Vilma.Kriaucioniene@lsmuni.lt (V.K.); janina.petkeviciene@lsmuni.lt (J.P.); 2 Clinic of Dental and Oral Pathology, Faculty of Odontology, Lithuanian University of Health Sciences, 50161 Kaunas, Lithuania; jolanta.siudikiene@lsmu.lt

**Keywords:** oral health, oral hygiene, socio-demographic factors, health behavior, dental care utilization, trends

## Abstract

This study aimed to examine 20-year trends (1994–2014) in self-reported oral health and oral hygiene and to assess the associated factors in a Lithuanian population aged 20–64 years. Nationally representative cross-sectional data on 8612 men and 11,719 women were obtained from 11 biennial postal surveys of Lithuanian health behavior monitoring. Dentate status was assessed by asking about the number of missing teeth. Over the study period, the proportion of men with all teeth increased from 17.5% to 23.0% and the same proportion increased in women—from 12.5% to 19.6%. The prevalence of edentulousness was 2.8% in 2014. The proportion of individuals brushing teeth at least twice a day increased from 14.6% to 31.9% in men and from 33.0% to 58.8% in women. Multivariate logistic regression analysis revealed that older age, lower education, living in rural areas, daily smoking, confectionary consumption (only in women), obesity, no visits to a dentist during the past year, toothache and brushing teeth less than twice a day increased the odds of missing six or more teeth. Efforts should be made to promote good oral hygiene habits, prevent and control behavioral risk factors and increase access to dental care among risk groups.

## 1. Introduction

Oral health is an extremely important component of general health and physical and mental well-being. A new definition of oral health developed by the FDI World Dental Federation states that oral health “reflects the physiological, social, and psychological attributes that are essential to the quality of life” [1]. Despite the effort to improve oral health in recent decades, oral diseases remain highly prevalent worldwide, being one of the main public health challenges [2]. In 2015, the number of people with untreated oral conditions reached 3.5 billion and disability-adjusted life years due to untreated caries, severe periodontal disease and tooth loss increased by 64% from 1990 [3]. The Lithuanian population is characterized by poor oral health. According to data of epidemiological studies, more than 90% of the middle-aged and elderly population in Lithuania had dental caries or periodontal disease [4,5]. The prevalence of dental caries among 18-year-old Lithuanian adolescents was 78.3% [6].

Socioeconomic factors are associated with oral health status. Individuals from lower income and education groups experienced poorer oral health and a higher burden of untreated oral diseases [7,8,9]. Socioeconomic differences in oral health may be related to differences in access to dental care because in many countries, a high proportion of dental care expenditure is out-of-pocket payments. In Lithuania, dental care is also only partially covered through the health insurance system [10]. Thus, dental care utilization depends on personal economic resources.

Good oral hygiene is crucial for oral health. Tooth brushing habits play an important role in the pathogenesis of oral diseases such as dental caries and periodontal diseases [11,12]. The proportion of individuals brushing teeth at least twice a day differs between countries and social groups being lower among men, older people and those with lower social status [13,14,15]. In a Lithuanian study, only 57.7% of the individuals aged 45–54 years and 53.1% aged 55–64 years brushed their teeth twice a day [4].

Some health behaviors such as dietary patterns and smoking have been found associated with oral health. Consumption of foods high in sugars increased caries prevalence and severity [16,17]. Many factors may influence this effect: the availability of sugar for bacterial digestion, the presence of acidogenic bacteria in the plaque on teeth, teeth susceptibility and time of sugar contact with the tooth surface [18]. Daily use of fluoride toothpaste reduced but did not eliminate the association between the amount of sugars intake and dental caries [16]. Other foods containing carbohydrates, for example, sandwiches, also were shown to be associated with dental caries [17]. On the other hand, a healthy diet rich in fresh fruits, greens and beans may reduce the prevalence of dental caries [19,20]. Unhealthy dietary patterns and low physical activity are the main causes of overweight and obesity. Some studies reported that the oral health of individuals with a higher body mass index (BMI) was worse compared with individuals with a lower BMI [21,22]. A recent meta-analysis indicated an association between tobacco smoking and dental caries [23]. In a Finnish cohort study, daily smoking was independently related to caries development [24].

Evaluation and monitoring of factors associated with oral health are important for the assessment of oral health care needs, preventive measures and oral health system priorities. So far, very little is known about associations of social and health behavior factors with oral health status in the Lithuanian population. Furthermore, no study analyzed the time trends in oral health and oral hygiene in the country. This study aimed to examine 20-year trends in self-reported oral health and oral hygiene and to assess the associated factors in a Lithuanian adult population.

## 2. Materials and Methods

### 2.1. Study Design and Sample

The data were obtained from 11 cross-sectional surveys of Lithuanian health behavior monitoring [25]. Since 1994, the postal surveys focusing on health-related behaviors, self-reported health status and the usage of health services have been carried out biennially. For every survey, a nationally representative simple random sample of the whole population aged 20–64 was drawn from the National Population Register. The sample consisted of 3000 individuals in each of the 1994–2008 surveys and 4000 in each of the 2010–2014 surveys. The questionnaires with one reminder were mailed between April and June. The overall response rates varied from 51.1% to 74%. In total, 8738 men and 11,822 women participated in the surveys (Table 1). Respondents with missing information for any of the study variables were excluded from the analytical sample (126 men and 103 women). Finally, data of 8612 men and 11,719 women were analyzed.

All surveys were approved by the Lithuanian Bioethics Committee (protocol No. 6B-10-61). The respondents signed an informed consent form for participation in the study.

### 2.2. Measurements

The dentate status was assessed by the question: “How many teeth are you missing?”, with five response options (none; 1–5 are missing; 6–10 are missing; over 10 but not all; all are missing—artificial teeth). The options were further categorized into two groups: (1) having all teeth or missing less than 6 teeth and (2) missing 6 or more teeth. Toothache was determined using the question: “Did you feel toothache during the past month?”, with answer options (1) yes, or (2) no. The use of dental care services was measured with the question: “How many times did you visit a dentist during the past 12 months?” Respondents were divided into three groups by the number of visits: no visit, 1–2 visits and 3 or more visits. Tooth brushing was inquired with the question: “How often do you brush your teeth?” Possible answer choices were: (1) more than once a day, (2) once a day, (3) not every day or (4) never. They were categorized into two groups: (1) brushing at least twice a day and (2) brushing less often or never.

Dentate status and tooth brushing were assessed concerning the socio-demographic variables such as age, education and place of residence. Age was analyzed in four groups: 20–34, 35–44, 45–54 and 55–64. The respondents were categorized into three groups according to the highest level of completed education: (1) low education (primary education, incomplete secondary education or secondary school), (2) intermediate education (vocational school) and (3) high education (college or university). According to the administrative classification of places of residence, the respondents were grouped as living in cities (capital city and four largest cities of Lithuania), towns (centers of municipalities and towns with at least 2000 inhabitants) and villages.

The associations of dentate status and tooth brushing with several health behaviors (smoking, consumption of strong alcoholic drinks (hard liquor), leisure-time physical activity, consumption of fresh vegetables and confectionary) were analyzed. All variables were dichotomized: current daily smokers and others (occasional smokers, quitters and never-smokers); individuals consuming strong alcoholic drinks at least once a week and consuming less frequently or never; respondents having leisure-time physical activity lasting at least half an hour on four and more days a week and exercising less frequently; daily consumers of fresh vegetables and those consuming less often or never; and individuals consuming confectionary at least 3 days a week and less often. Self-reported weight and height were used to calculate body mass index (BMI). Overweight was defined as BMI 25–29 kg/m^2^, and obesity as BMI ≥ 30 kg/m^2^.

### 2.3. Statistical Analysis

Data were analyzed using the statistical package IBM SPSS Statistics for Windows, Version 20.0 (IBM Corp.: Armonk, NY, USA, released 2011). The categorical variables were presented as proportions and compared using a χ^2^ test and *z*-test with Bonferroni correction. Secular trends in proportions of missing teeth and brushing teeth at least twice a day between 1994 and 2014 were tested using linear regression analysis with the corresponding proportion as the dependent variable and the survey year as the predictor. Beta coefficients showed biannual changes in dentate status and proportion of frequent teeth brushing.

Multivariate logistic regression analysis was used to assess the associations of dentate status and tooth brushing with socio-demographic and health behavior variables as well as the use of dental care. Data of men and women were analyzed separately. For all models, the Hosmer and Lemeshow test was not significant (*p* > 0.05), indicating that the data fit the models well.

*p* values of less than 0.05 were considered to indicate statistical significance.

## 3. Results

The characteristics of the study population are presented in Table 2. More women than men were highly educated and lived in cities and towns. A significantly higher proportion of men than women were daily smokers and consumed strong alcoholic drinks at least once a week. Men consumed fresh vegetables and confectionary less often and were more physically active during leisure time compared with women. The prevalence of overweight was higher in men, while more women than men were with obesity.

Almost a half (49.5%) of dentate women and a quarter (24.7%) of men answered that they were brushing teeth at least twice a day. More men than women indicated that they had suffered from toothache during the past month, at 15.6% and 12.4%, respectively. Women more often than men reported that they visited a dentist during the past year. A slightly higher proportion of men, compared with women, indicated missing 1–5 teeth, while more women than men were missing 6 and more teeth.

From 1994 to 2004, the proportion of men with all teeth varied, however, it did not change significantly (Table 3). Since 2004, an increasing tendency in the proportion of men having all teeth was observed (an increase by 0.836% biennially). Over the study period, the increase in the proportion of women with all teeth was more stable than in men. This proportion increased from 12.5% to 19.6% (an increase of 0.945% biennially). The proportion of men missing 1–5 teeth decreased from 54.2% in 1994 to 44.6% in 2014 (a decrease of 0.945% biennially); however, the proportion of edentulous men slightly increased. The significant decrease was found in the proportion of women missing 6 or more teeth (a decrease of 0.875% biennially).

**Figure 1:**
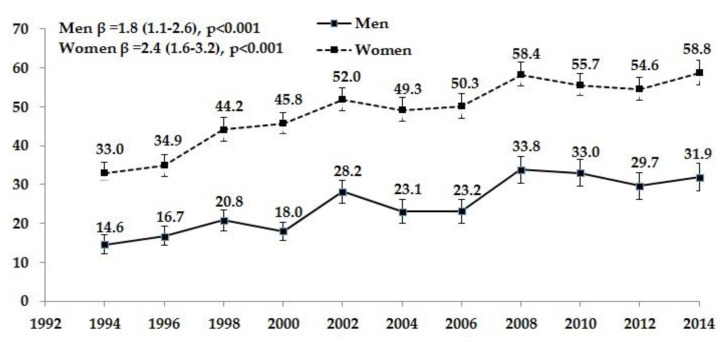
Proportion (%) of men and women brushing teeth at least twice a day in 1994–2014 (edentulous individuals are excluded).

Between 1994 and 2014, the proportion of women brushing teeth at least twice a day increased from 33.0% to 58.8% (an increase of 2.4% biennially) (Figure 1.). Over the study period, the same proportion of men more than doubled from 14.6% to 31.9% (an increase of 1.8% biennially); however, it remained much lower compared with women.

Logistic regression analysis of the associations between brushing teeth at least two times a day and socio-demographic factors revealed that the odds are decreasing with age, especially in dentate men (Table 4). In the oldest age group (55–64 years), the likelihood of brushing teeth at least twice a day was lower by 38% in men and by 23% in women, if compared with the youngest age group (20–34 years). Highly educated individuals and city inhabitants had higher odds of regular teeth brushing than men and women with lower education and living in towns and villages.

Unhealthy behaviors decreased the likelihood of brushing teeth at least twice a day. Current daily smoking was associated with 37% lower odds of regular teeth brushing practice in men and 18% in women. Men consuming strong alcoholic drinks at least once a week were less likely to brush their teeth twice a day. In men, leisure-time physical activity at least on four days a week was associated with higher odds of regular teeth brushing. Men and women consuming fresh vegetables daily and women eating confectionary less often than three days a week were more likely to brush their teeth twice a day. Men and women with obesity and women with overweight had worse oral hygiene habits than those with normal BMI. Obesity decreased the odds of regular teeth brushing by 26% in men and 51% in women. Respondents visiting a dentist had higher odds of brushing teeth twice a day than those who reported no visits. An inverse association was found between toothache and frequent teeth brushing in women. The likelihood of healthy oral hygiene habits decreased with the increase in the number of missing teeth. Men missing 6 or more teeth had, by 59% and women by 47%, lower odds of brushing teeth twice a day as compared with respondents with all teeth.

Nagelkerke’s R^2^ showing how much variation in frequent tooth brushing is explained by the model was 0.177 for men and 0.153 for women.

The results of the logistic regression analysis for associations between missing 6 or more teeth with the socio-demographic and health behavior factors are presented in Table 5. In both genders, the likelihood of missing 6 or more teeth increased with age (by 38.8 times in men and 31.1 times in women, if compared with the oldest and the youngest age groups). Higher education was associated with better oral health. Men and women with a high level of education were less likely to miss 6 or more teeth, respectively by 44% and 57%, compared with low-educated respondents. Rural inhabitants had a worse oral health situation than respondents living in cities. Daily smoking increased the odds of missing 6 and more teeth by 78% in men and 52% in women. Frequent consumption of confectionary (at least three times a week) was associated with higher odds of missing 6 and more teeth only in women. Consumption of strong alcoholic drinks, leisure-time physical activity and fresh vegetable consumption were not associated with the analyzed variable. Women with overweight and obesity were more likely to miss 6 and more teeth than women with a normal BMI. Unexpectedly, men with overweight had lower odds of missing 6 or more teeth compared with men with normal weight.

The significantly lower likelihood of missing 6 or more teeth had respondents who visited a dentist 1–2 times during the past year compared with those not visiting a dentist. More frequent visits to a dentist were not associated with the analyzed number of missing teeth. Suffering from toothache increased the likelihood of missing 6 or more teeth. Odds of missing 6 or more teeth were lower by 77% in men and by 46% in women brushing their teeth less often than twice a day.

Nagelkerke’s R^2^ for the logistic regression model of men was 0.330 and women 0.346, which suggests that the model explains more than 30% of the variation in the proportion of missing 6 teeth or more.

Logistic regression analysis for associations between missing 10 or more teeth and the same independent variables as in Table 5 showed very similar results, plus more statistically significant results in women (data are not shown). The likelihood of missing 10 or more teeth was lower in women consuming fresh vegetables daily than consuming less often (OR 0.77; CI 0.65–0.91; *p* = 0.003) and visiting a dentist three or more times during the past year than having no visit (OR 0.72; CI 0.60–0.86; *p* < 0.001). All other associations were the same as for missing 6 or more teeth.

## 4. Discussion

The WHO recognizes a high prevalence of oral diseases as an important public health problem due to their associations with other chronic diseases such as cardiovascular disease, diabetes and cancer, strong influences on people’s well-being and high economic costs [26]. A limited number of previous cross-sectional studies carried out in Lithuania demonstrated a poor oral health situation in children and adults [4,5,6]. However, no study was conducted at the national level using large samples. Our study is the first to examine 20-year trends in self-reported oral health and oral hygiene in a nationally representative sample of a Lithuanian adult population. The results demonstrated the positive trend in the proportions of individuals having all teeth and brushing teeth at least twice a week. Dentate status was slightly better in men, while a higher proportion of women brushed their teeth regularly. Oral health and oral hygiene inequalities between age and education groups as well as by place of residence were identified. The associations of oral health and oral hygiene with socio-demographic and health behavior factors as well as dental care utilization were studied. Unhealthy diet, obesity and smoking were associated with worse dentate status and oral hygiene. Suffering from toothache and no visit to a dentist increased the likelihood of poor oral health.

Frequent loss of permanent teeth was demonstrated by other authors who have evaluated self-reported oral health [13,27,28]. In the Baltic country Estonia, where the study was carried out using the same methodology as in our study, only 29.5% of men and 30.3% of women reported retention of all permanent teeth [27]. Edentulism was disclosed by 2.1% of individuals. A study carried out in Portugal found that 70.3% of respondents had lost at least one permanent tooth and 32.5% more than six permanent teeth [13]. In line with our results, this study revealed a higher number of missing teeth in women than men. We found only a small difference in the dentate status of men and women; however, these results are difficult to explain. Compared with men, Lithuanian women had healthier oral hygiene habits, visited a dentist more frequently and less often reported toothache during the past month. A lower proportion of women smoked daily and consumed strong alcoholic drinks frequently, while a higher proportion consumed fresh vegetables daily. Only frequent confectionary consumption was more prevalent among women than men. Hence, the causes of tooth loss in women should be further investigated.

Older people tend to have more oral health problems than younger ones. In our study, the likelihood of missing at least six teeth was more than 30 times higher in individuals 55–64 years old than in those 20–34 years old. In the USA, 67% of adults aged 20–39 had retained all of their permanent teeth compared with 34% of adults aged 40–64 [29]. A steep increase in the loss of teeth with age was also reported by other authors [13,27,28,30]. The tooth loss in adults was associated with chewing problems, lower diet quality, reduced nutrient intake and low serum albumin levels [31,32].

A lot of studies revealed that the prevalence of oral diseases is associated with socioeconomic status [7,9,33]. Education is the most common indicator used for the evaluation of socioeconomic differences in oral health. Our data are consistent with previous results showing that a lower level of education increases the risk of tooth loss [13,27,33,34]. In Lithuania, men and women with higher levels of education had an almost twice lower likelihood for the loss of six or more teeth than individuals with incomplete secondary or secondary education. Furthermore, Lithuanians living in villages were more likely to lose their teeth than city inhabitants. A study carried out in Switzerland found that the population in rural and urban areas had similar numbers of missing teeth; however, older individuals from rural regions have lost more teeth than those from cities [28]. Previous research has shown a strong association between socioeconomic status and the use of dental care [35]. Out-of-pocket payments comprise a significant proportion of dental care costs. In Lithuania, the National Health Insurance Fund only partly covers the dental care cost provided in public facilities or by private dentists contracted with the Fund [10]. People with lower financial resources may lack access to high-quality care. The preceding publication from a Lithuanian health behavior monitoring study reported a strong positive association between education and number of visits to a dentist [36]. According to our data, number of visits to a dentist was associated with number of missing teeth. Lower odds of missing six or more teeth were identified for respondents who visited a dentist one–two times during the last year compared with those not visiting a dentist.

Our study also found that the loss of teeth is associated with toothbrushing habit. These findings are in line with other studies that reported tooth loss as a result of poor oral hygiene causing caries and periodontal diseases [11,12,13,14,37,38]. Our data showed the increasing 20-year trend in the proportion of men and women brushing teeth at least twice a day. The gradual increase in the frequency of regular toothbrushing during 40 years was reported by Swedish authors [39]. Education for oral hygiene since kindergarten and primary school in recent decades might have an effect on positive trends in toothbrushing habit in today’s adult population. In our study, the lower brushing frequency was identified in men, lower educated, living in rural areas, having unhealthy behaviors and obese individuals. Other studies demonstrated similar associations between oral hygiene and socio-demographic as well as health behavior factors [13,14,15,37,38]. A large Scottish study revealed that individuals who brushed their teeth less often than twice a day were more likely to be men, slightly older and of lower social status: they had a higher prevalence of unhealthy behaviors such as smoking, physical inactivity and obesity [14]. The same factors were associated with the number of missing teeth. We used multivariate logistic regression analysis to demonstrate an independent effect of the analyzed factors on oral health status.

In our study, unhealthy behaviors were associated with poor oral health. Smokers had higher odds for missing teeth than non-smokers. Other authors found that daily smoking was related to caries development [23,24,40]. Further, smokers were more likely to have the severe periodontal disease [40]. A recent study demonstrated the impact of nicotine on oral microorganisms which was related to increased risk of dental caries [41]. The effect of sugars on dental caries development is well known [16,17,18]. An experimental study demonstrated that the caries process was related to the ability of sugars to regulate oral microecology [42]. Sucrose supplementation disrupted the homeostasis between acid-producing and alkali-producing bacteria. The importance of the frequency of sugar consumption over the amount was shown in some studies [18,43]. In an 11-year follow-up study, poor diet predicted periodontal disease development [44]. Our findings demonstrated the associations between frequency of confectionary consumption and oral health in women.

Previous studies identified a positive association between dental caries and being overweight or obese [21,22,38]. The prevalence of periodontal disease was also higher in individuals with obesity [38,45]. Our study confirmed the association between oral health and BMI, showing higher odds of missing six or more teeth for individuals with overweight. One of the explanations of the association between overweight and oral health might be the high consumption of sugars which may cause an increase in BMI and dental caries development. Australian researchers demonstrated that the statistical significance between dental caries and being overweight or obese disappeared after adjustment for sugar consumption [46].

Our study has some strengths and limitations. The strengths of our study include the usage of nationally representative data collected following the same methodology over 20 years. The same questionnaires were used in all surveys ensuring comparability of data. Several limitations also should be mentioned. All data were self-reported including oral health measures. Some studies revealed that the self-reported number of teeth agreed closely with the corresponding clinical measure [47,48]. Moreover, we were not able to identify other causes than the oral diseases of missing teeth (trauma, agenesis, etc.). The study design was cross-sectional; therefore, only associations, not causal links, can be established. Response rates declined across the survey years. In general, respondents tend to have higher socioeconomic status and report better health and health behavior than non-respondents. However, several studies proved that non-response may bias the prevalence, though this does not have any statistically significant effect on associations between variables [49,50].

## 5. Conclusions

Over 20 years, oral health status and oral hygiene habits have improved in the Lithuanian adult population. Older age, lower education, living in rural areas, daily smoking, confectionary consumption (only in women), obesity, no visits to a dentist during the past year, toothache and brushing teeth less than twice a day are significant predictors of missing teeth. Our findings provide the evidence that efforts should be made to promote good oral hygiene habits, prevent and control behavioral risk factors and increase access to dental care among risk groups.

## Figures and Tables

**Table 1:** Participants of a Lithuanian health behavior monitoring study, 1994–2014.

Study Year	Men *n* (%)	Women *n* (%)	Total (*n*)
1994	787 (42.2)	1077 (57.8)	1864
1996	920 (45.5)	1101 (54.5)	2021
1998	823 (43.9)	1051 (56.1)	1874
2000	996 (45.4)	1199 (54.6)	2195
2002	836 (44.4)	1047 (55.6)	1883
2004	784 (43.0)	1038 (57.0)	1822
2006	723 (41.6)	1016 (58.4)	1739
2008	737 (41.8)	1026 (58.2)	1763
2010	752 (37.7)	1245 (62.3)	1997
2012	725 (40.3)	1076 (59.7)	1801
2014	655 (40.9)	946 (59.1)	1601
Total	8738 (42.5)	11822 (57.5)	20560

**Table 2:** Characteristics of the study population (%).

Characteristic	Men *n* = 8612	Women *n* = 11719	*p*-Value
Age groups			0.011
20–34	31.9	30.4 *	
35–44	24.3	23.8	
45–54	23.6	23.9	
55–64	20.2	21.9 *	
Education			<0.001
Low	45.2	35.8 *	
Intermediate	35.7	37.3 *	
High	19.1	26.9 *	
Place of residence			<0.001
Cities	42.1	45.5 *	
Towns	26.9	28.6 *	
Villages	31.0	25.9 *	
Daily smoking			<0.001
Yes	41.9	12.6	
No	58.1	87.4	
Strong alcohol consumption at least ones a week			<0.001
Yes	28.6	8.9	
No	71.4	91.1	
Leisure-time physical activity			<0.001
≥4 days/week	24.9	22.2	
<4 days/week	75.1	77.8	
Daily fresh vegetable consumption			<0.001
Yes	16.3	23.3	
No	83.7	76.7	
Confectionary consumption			<0.001
≥3 days/week	25.5	28.1	
<3 days/week	74.5	71.9	
Body mass index			<0.001
Normal	45.4	52.3 *	
Overweight	39.5	29.0 *	
Obesity	15.0	18.7 *	
Toothache during past month			<0.001
Yes	15.6	12.4	
No	84.4	87.6	
Visits to a dentist during the past year			<0.001
No visit	46.6	28.4 *	
1–2 visits	34.4	42.0 *	
3 and more visits	19.0	29.6 *	
Tooth brushing **			<0.001
At least twice a day	24.7	49.5	
Once a day	42.6	39.3	
Less often	32.6	11.2	
Number of teeth			<0.001
All teeth	17.9	16.4 *	
Missing 1–5 teeth	50.7	48.1 *	
Missing 6–10 teeth	16.0	17.7 *	
Missing >10 teeth, but have some	12.9	15.3 *	
Edentulous	2.5	2.5	

* *p* < 0.05 compared with men (*z* test with Bonferroni correction); ** edentulous individuals are excluded.

**Table 3:** Distribution (%) of men and women by a number of teeth in 1994–2014.

Study Year	Men *n* = 8612				Women *n* = 11719			
	All Teeth	Missing 1–5 Teeth	Missing ≥6 Teeth	Edentulous	All Teeth	Missing 1–5 Teeth	Missing ≥6 Teeth	Edentulous
1994	17.5	54.2	26.4	1.9	12.5	45.9	38.2	3.3
1996	14.3	53.2	30.2	2.3	9.8	47.8	39.3	3.1
1998	12.7	55.4	30.0	2.0	13.4	50.7	33.7	2.2
2000	15.4	53.1	30.1	1.4	13.6	49.5	35.1	1.8
2002	19.2	49.6	29.5	1.7	17.6	50.0	30.0	2.3
2004	15.3	50.4	31.3	3.0	17.4	48.0	32.3	2.3
2006	17.1	49.6	30.6	2.7	18.4	48.9	30.2	2.6
2008	23.1	51.8	22.1	3.1	19.9	44.8	32.8	2.5
2010	21.5	44.7	30.4	3.4	19.2	47.7	30.9	2.2
2012	20.5	47.9	27.2	4.3	19.2	47.0	30.7	3.1
2014	23.0	44.6	29.6	2.8	19.6	49.2	28.4	2.8
β *	0.836	−0.945	−0.088	0.192	0.945	−0.056	−0.875	−0.007
*p*-value *	0.005	0.001	0.749	0.008	<0.001	0.757	0.001	0.880

* Linear regression analysis (β—regression coefficient, *p*—the level of statistical significance, that β differs from 0).

**Table 4:** Odds ratios * and 95% confidence intervals for brushing teeth at least twice a day.

Risk Factor	Brushing Teeth at Least Twice a Day					
	Men			Women		
	OR	95% CI	*p*-Value	OR	95% CI	*p*-Value
Age groups						
20–34	1			1		
35–44	0.82	0.70–0.98	0.025	0.95	0.83–1.09	0.477
45–54	0.75	0.62–0.91	0.003	0.97	0.84–1.13	0.70
55–64	0.62	0.50–0.79	<0.001	0.77	0.65–0.91	0.003
Education						
Low	1			1		
Intermediate	1.19	1.02–1.38	0.023	1.37	1.23–1.54	<0.001
High	2.00	1.70–2.35	<0.001	2.07	1.83–2.34	<0.001
Place of residence						
Cities	1			1		
Towns	0.83	0.71–0.96	0.014	0.79	0.71–0.88	<0.001
Villages	0.53	0.45–0.62	<0.001	0.60	0.53–0.68	<0.001
Daily smoking						
No	1			1		
Yes	0.63	0.55–0.72	<0.001	0.82	0.71–0.94	0.004
Strong alcohol consumption at least ones a week						
No	1			1		
Yes	0.86	0.75–0.99	0.043	0.88	0.75–1.03	0.099
Leisure-time physical activity						
<4 days/week	1			1		
≥4 days/week	1.31	1.14–1.52	<0.001	0.97	0.87–1.09	0.654
Daily fresh vegetable consumption						
No	1			1		
Yes	1.61	1.38–1.87	<0.001	1.47	1.32–1.64	<0.001
Confectionary consumption						
<3 days/week	1			1		
≥3 days/week	1.03	0.89–1.19	0.673	0.79	0.71–0.88	<0.001
Body mass index						
Normal	1			1		
Overweight	1.09	0.94–1.25	0.255	0.72	0.64–0.81	<0.001
Obesity	0.74	0.60–0.90	0.003	0.49	0.43–0.57	<0.001
Toothache during past month						
No	1			1		
Yes	0.89	0.74–1.07	0.215	0.81	0.70–0.94	0.006
Visits to a dentist during the past year						
No visit	1			1		
1–2 visits	1.70	1.47–1.96	<0.001	1.50	1.33–1.68	<0.001
3 and more visits	1.73	1.46–2.05	<0.001	1.59	1.40–1.80	<0.001
Number of teeth						
All teeth	1			1		
Missing 1–5 teeth	0.67	0.57–0.79	<0.001	0.72	0.63–0.83	<0.001
Missing 6 or more teeth	0.41	0.33–0.51	<0.001	0.53	0.44–0.62	<0.001
Study year	1.04	1.03–1.05	<0.001	1.03	1.03–1.04	<0.001

*—edentulous individuals are excluded. Abbreviations: OR—odds ratio; CI—confidence interval.

**Table 5:** Odds ratios and 95% confidence intervals for missing 6 teeth or more.

Risk Factor	Missing 6 Teeth or More					
	Men			Women		
	OR	95 CI	*p*-Value	OR	95 CI	*p*-Value
Age						
20–34	1			1		
35–44	6.43	5.03–8.22	<0.001	5.62	4.59–6.88	<0.001
45–54	18.11	14.19–23.11	<0.001	16.32	13.32–20.00	<0.001
55–64	38.83	29.91–50.40	<0.001	31.05	24.95–38.64	<0.001
Education						
Low	1			1		
Intermediate	0.82	0.70–0.95	0.009	0.79	0.69–0.90	0.001
High	0.56	0.46–0.68	<0.001	0.43	0.37–0.50	<0.001
Place of residence						
Cities	1			1		
Towns	1.05	0.89–1.24	0.558	1.30	1.13–1.48	<0.001
Villages	1.21	1.03–1.43	0.024	1.52	1.31–1.76	<0.001
Daily smoking						
No	1			1		
Yes	1.78	1.54–2.05	<0.001	1.52	1.28–1.80	<0.001
Strong alcohol consumption at least ones a week						
No	1			1		
Yes	1.05	0.90–1.21	0.545	1.18	0.97–1.43	0.090
Leisure-time physical activity						
<4 days/week	1			1		
≥4 days/week	1.11	0.95–1.29	0.201	1.00	0.88–1.14	0.998
Daily fresh vegetable consumption						
No	1			1		
Yes	0.91	0.76–1.09	0.308	0.925	0.81–1.06	0.245
Confectionary consumption						
<3 days/week	1			1		
≥3 days/week	0.89	0.76–1.05	0.171	1.17	1.03–1.33	0.020
Body mass index						
Normal	1			1		
Overweight	0.85	0.73–0.99	0.036	1.28	1.12–1.47	<0.001
Obesity	1.05	0.86–1.27	0.634	1.66	1.42–1.94	<0.001
Toothache during past month						
No	1			1		
Yes	1.37	1.13–1.66	0.001	1.32	1.11–1.58	0.002
Visits to a dentist during the past year						
No visit	1			1		
1–2 visits	0.65	0.56–0.76	<0.001	0.63	0.55–0.73	<0.001
3 and more visits	1.17	0.97–1.40	0.095	0.99	0.85–1.15	0.886
Tooth brushing						
At least twice a day	1			1		
Less often or never	1.77	1.48–2.10	<0.001	1.46	1.30–1.65	<0.001
Study year	0.99	0.98–1.01	0.196	0.97	0.96–0.98	<0.001

Abbreviations: OR—odds ratio; CI—confidence interval.
